# Masculinities and Aging in Ireland

**DOI:** 10.1093/geroni/igaa057.3017

**Published:** 2020-12-16

**Authors:** Aine Ni Leime

**Affiliations:** NUI Galway, Galway, Galway, Ireland

## Abstract

There is a need for research to gain understanding of the social and cultural constructions of ageing masculinities that, as Gullette emphasises, operate together to construct a ‘culture of decline’. This presentation explores how cultural images of older men inform constructions of ageing and lived realities in Ireland. It draws on the Irish findings from a cross-national, inter-disciplinary project conducted in 2019 investigating older men’s perceptions of how they are represented in film and advertising. It applies innovative narrative and thematic analysis to data from four focus group discussions, interviews and reflective diaries, to explore participants’ (Irish men aged 65+) reactions to the portrayal of older men in TV and film. Stereotypes identified included older men as conservative, grumpy, sad, streetwise, trickster, or action hero. Thematic analysis identified themes including men’s identification with their jobs; their diminishing roles in the family; and old age as a matter of perception.

